# Dynamic network biomarker C1QTNF1 regulates tumor formation at the tipping point of hepatocellular carcinoma

**DOI:** 10.17305/bb.2024.10103

**Published:** 2024-08-01

**Authors:** Haoyuan Yu, Xijing Yan, Guanxing Chen, Rong Li, Zhou Yang, Zhixing Liang, Linsen Ye, Yunhao Chen, Yang Li

**Affiliations:** 1 Department of Hepatic Surgery and Liver Transplantation Center, The Third Affiliated Hospital of Sun Yat-sen University, Guangzhou, China; 2 Guangdong Provincial Key Laboratory of Liver Disease Research, Guangzhou, China; 3 Artificial Intelligence Medical Research Center, School of Intelligent Systems Engineering, Sun Yat-sen University, Shenzhen, China; 4 Biotherapy Center, The Third Affiliated Hospital of Sun Yat-sen University, Guangzhou, China

**Keywords:** Hepatocellular carcinoma (HCC), Dynamic Network Biomarker (DNB), C1q and tumor necrosis factor (TNF)-related 1 (C1QTNF1), diagnosis, prognosis

## Abstract

Identifying the precise moment before the onset of hepatocellular carcinoma (HCC) remains a significant challenge in the medical field. The existing biomarkers fall short of pinpointing the critical point preceding HCC formation. This study aimed to determine the exact tipping point for the transition from cirrhosis to HCC, identify the core Dynamic Network Biomarker (DNB), and elucidate its regulatory effects on HCC. A spontaneous HCC mouse model was established to mimic HCC formation in patients with chronic hepatitis. Using the DNB method, C1q and tumor necrosis factor (TNF)-related 1 (C1QTNF1) protein was identified as the key DNB at the crucial tipping time of spontaneous HCC development. Both in vitro and in vivo studies showed that C1QTNF1 could inhibit tumor growth. Overexpression of C1QTNF1 before the tipping point effectively prevented HCC occurrence. Patients with elevated C1QTNF1 expression demonstrated improved overall survival (OS) (*P* ═ 0.03) and disease-free survival (DFS) (*P* ═ 0.03). The diagnostic value of C1QTNF1 was comparable to that of alpha-fetoprotein (AFP) (area under the curve [AUC] ═ 0.84; sensitivity 85%; specificity 80%). Furthermore, our research indicated that platelet-expressed C1QTNF1 is involved in cancer-associated signaling pathways. Our findings introduce a novel perspective by highlighting C1QTNF1 as the pivotal biomarker at the tipping point of primary HCC formation using DNB. We propose C1QTNF1 as a prognostic biomarker for HCC, potentially influencing tumor development through a platelet-related cancer signaling pathway.

## Introduction

Hepatocellular carcinoma (HCC) is ranked as the seventh most common type of cancer and the third leading cause of cancer-related mortality worldwide [1–3]. The comprehensive treatment for HCC encompasses a variety of methods, including surgical resection, liver transplantation, chemotherapy, targeted drug therapy, radiation therapy, and interventional therapy. In addition, emerging therapies, such as immunotherapy and targeted immunotherapy continue to evolve and improve [4–6]. Exploring potential molecular mechanisms that can lead to the development of HCC is essential, as it aids in preventing the formation of HCC and devising therapeutic strategies. The diagnosis and screening of HCC often rely on alpha-fetoprotein (AFP), which is widely utilized as a serum biomarker [7]. Nevertheless, the usability of this biomarker is limited due to its sensitivity and the restricted range of incremental concentrations. Consequently, there is an urgent need to enhance clinical outcomes by developing dependable and innovative diagnostic biomarkers [8, 9].

Many intricate systems experience a crucial shift prior to undergoing a transformation, abruptly transitioning into an entirely distinct state [10]. A so-called tipping point exists, marking the juncture at which significant or fundamental changes may occur. Extensive focus has been given to the identification of tipping points in various systems, including but not limited to social ecology, finance, and climate systems. Diverse biological processes have also exhibited comparable significant state changes. Certain long-term illnesses might experience a slow progression that spans over several years or even decades prior to experiencing a severe decline. Cell differentiation is also considered a key transition in embryonic development. Gaining an understanding of the mechanisms behind disease progression or embryonic development heavily relies on the identification of these significant shifts or pivotal moments in biological systems [11]. Typically, the development of intricate biological systems is regarded as a dynamic system that varies over time and is nonlinear in nature [12]. From the perspective of dynamical systems, a biological process can be divided into three phases: (1) the initial state before the transition, which is a stable condition characterized by high flexibility; (2) the critical or tipping point, an unstable phase with limited susceptibility to disruptions prior to the qualitative alteration; and (3) the subsequent state after the transition, which is another stable condition characterized with significant flexibility following the alteration [13]. Conventional biomarkers, employed for discriminating tumor samples from normal samples or for detecting disease conditions, rely on the dissimilar expression data of molecules between normal and diseased states. However, these measures are not applied during the pre-illness phase or at the critical moment, which is why traditional indicators may fail to anticipate the onset of illness [14]. The detection of crucial tipping points in biological systems has been accomplished using a recently suggested approach known as Dynamic Network Biomarker (DNB) [15, 16]. Specifically, as a system nears a critical state, a prominent set of highly variable factors arises and demonstrates a significant collaborative impact on molecular connections. This phenomenon can be utilized to accurately assess the stability and critical nature of a system on a network scale [17].

Based on a spontaneous HCC mouse model, we examined time-series gene expression utilizing a DNB method. The analysis identified C1q and tumor necrosis factor (TNF)-related 1 (C1QTNF1) protein as the core member during the critical period of HCC progression. C1QTNF1 belongs to the C1QTNF family, a family of adipokines recognized for their potential roles in metabolism and immunity [18, 19]. Through both in vitro and in vivo experiments, we discovered that C1QTNF1 possesses the ability to inhibit tumor growth. Furthermore, we validated the diagnostic and prognostic significance of C1QTNF1. Additional analysis revealed a significant correlation between platelets and the expression of C1QTNF1. Collectively, these findings offer fresh perspectives in recognizing the role of C1QTNF1 as the crucial factor at the tipping point of primary HCC development through DNB analysis. These results serve as a foundation for future investigations aimed at identifying novel therapeutic targets.

## Materials and methods

### Animal studies

Male mice (C57BL/6), aged four weeks, were randomly divided into eight groups, with each group consisting of four mice. These mice received an intraperitoneal injection of 30 mg kg^−1^ N-nitrosodiethylamine (DEN) (Cat: N0258, Sigma Aldrich, USA) [20]. Liver samples from mice in each group were collected monthly, from the first to the eighth month following the injection (groups A–H represent the first to eighth months, respectively). Histological analyses were performed on both the HCC tumor tissues and adjacent non-tumor tissues. The samples were frozen at –80 ^∘^C for subsequent utilization.

Six-week-old male nude mice were randomly divided into two groups, each consisting of five mice. These nude mice were subcutaneously implanted with 1×10^6^ tumor cells in the right groin area. SNU449 cells, either transfected with Lentivirus scramble or engineered to overexpress C1QTNF1, were injected into the mice. Tumor volume was measured every other day, and after 16 days, the tumors were harvested.

### Cell culture and transfection

An immortalized human hepatocyte liver cell line (THLE-2) and human hepatoma cell lines (SNU398 and SNU449) were obtained from the American Type Culture Collection (ATCC). Additionally, human hepatoma cell lines (Huh7 and Hep3B) and a mouse hepatoma cell line (Hepa1-6) were sourced from the Type Culture Collection of the Chinese Academy of Sciences (Shanghai, China). The cells were cultured in high-glucose Dulbecco’s Modified Eagle’s Medium (DMEM) (Gibco, USA) supplemented with 10% fetal bovine serum (FBS) (Gibco, USA) and incubated at 37 ^∘^C in an atmosphere containing 5% CO_2_. C1QTNF1 overexpression was achieved through transfection with a lentivirus construct provided by JiKai Co., Ltd.

### Transwell invasion assay

For the migration assay, cells were resuspended in serum-free DMEM and placed in the upper chamber. DMEM containing 20% FBS was added to the lower chamber. For the invasion assay, the upper chamber membrane was pre-coated by adding 40 µL of Matrigel to its bottom. Following a 24-h incubation period for the migration assay or a 48-h incubation period for the invasion assay, the cells that adhered to the lower chamber were immobilized using 4% paraformaldehyde for 20 min. Subsequently, these cells were stained with crystal violet.

### Wound-healing scratch assay

Cells were cultured in 6-well plates at a density of 2 × 10^6^/well in a growth medium until they reached confluence. Subsequently, a scratch wound was made at the center at the bottom of each well using a pipette tip. The initial scratch was immediately photographed, and subsequent photographs were taken at 12-h intervals. The healing ratio at 24 h was determined by comparing the width of the wound at 24 h with the initial wound width at 0 h.

### The clonogenic assay

Cells overexpressing C1QTNF1 and those serving as the negative control were seeded in 6-well plates at a density of 1000 cells per well. Following incubation in an atmosphere of 5% CO_2_ at 37 ^∘^C for two weeks, the cells were fixed with 4% paraformaldehyde for 30 min and subsequently stained with 0.1% crystal violet for 20 min. This experiment was repeated three times independently.

### Immunohistochemistry

Immunohistochemical analysis was performed on a total of 96 paraffin-embedded HCC samples. The tissue specimens were obtained from HCC patients who underwent hepatectomy at the Third Affiliated Hospital of Sun Yat-sen University between January 2013 and January 2021. The tissue sections underwent dewaxing and hydration processes, followed by antigen retrieval using ethylenediaminetetraacetic acid (EDTA) at a pH of 8.0. To calculate immunochemical scores, the staining intensity and the count of positive cells were assessed and multiplied together. The scores for staining intensity ranged from 0 to 3 and the number of positive cells ranged from 1 to 4. Scores below 6 were deemed indicative of low expression, while scores of 7 or above were regarded as high expression.

### Immunofluorescent staining

Immunofluorescence (IF) staining was performed as previously described [21]. Initially, slides were dewaxed and hydrated, followed by antigen retrieval using EDTA at a pH of 8.0. Subsequently, the slides were treated with 0.5% Triton X-100 for 5 min. Blocking was performed using 1% bovine serum albumin for 30 min. The slides were then stained with anti-C1QTNF1 (sc-81943, Santa Cruz, 1:50 dilution) and anti-cluster of differentiation 41 (CD41) (ab134131, Abcam, 1:300 dilution) antibodies to detect C1QTNF1 and CD41, respectively. The slides were washed and incubated with cyanine 3 (Cy3)-conjugated goat anti-mouse IgG and fluorescein isothiocyanate (FITC)-conjugated goat anti-mouse IgG. Nuclei were stained with 4′, 6-diamidino-2-phenylindole (DAPI).

### Enzyme-linked immunosorbent assay (ELISA)

ELISA was performed using the Human C1QTNF1 ELISA kit (Novus). In brief, plasma samples were isolated from HCC patients and chronic hepatitis B (CHB) patients. Both the reference standard reagent and the plasma samples were then added to the 96-well plates, which were precoated with a specific antibody, and incubated at 37 ^∘^C for 90 min. After this incubation period, the liquid was discarded, and a bio-antibody was added to each well, followed by another incubation at 37 ^∘^C for 1 h. Subsequent to discarding the liquid and washing the wells, streptavidin-horseradish peroxidase (HRP) was introduced into each well and incubated at 37 ^∘^C for 30 min. The substrate regent was then introduced to evade the light. Following this, a stop solution was added to each well and the absorbance was measured at a wavelength of 450 nm.

### Immunoblotting

Western blot analyses were performed on either tumor tissue or corresponding paracancerous tissue from humans or mice, as described in previous methods [22]. Briefly, the procedure began with the separation of protein aliquots through sodium dodecyl sulfate-polyacrylamide gel electrophoresis (SDS-PAGE) using either 10% or 12.5% gels. The proteins were then transferred to polyvinylidene difluoride (PVDF) membranes (0.45 µm, Merck Millipore). After the transfer, the membranes were incubated with 5% bovine serum albumin in 0.1% tris-buffered saline with Tween 20 (TBST) for 1 h. This was followed by an overnight incubation at 4 ^∘^C with specific primary antibodies: anti-C1QTNF1 (ab25973, Abcam, 1:1000), CD41 (ab134131; Abcam, 1:1000), and anti-glyceraldehyde 3-phosphate dehydrogenase (GAPDH) (5174, CST, 1:1000) serving as an internal control. Subsequently, the protein expression levels were normalized to the GAPDH band density. These experiments were repeated with cells at least three times or with samples from a minimum of three different donors.

### Dynamic Network Biomarker (DNB) analysis

To identify the critical state preceding tumor transition and to pinpoint predictive biomarkers at the tipping point, we formulated a mathematical model using the DNB method [14, 16]. We defined a dominant group of molecules or genes that exhibit specific characteristics as the system approaches the critical state, according to three criteria: (1) noticeably increased standard deviations (SD*_i_*) of molecules within this dominant group; (2) substantially increased Pearson correlation coefficients (PCC*_i_*) among the expression levels within this dominant group; and (3) considerably decreased PCC (PCC*_o_*) between the molecules within this group and others. The quantification of these criteria into a single metric, the criticality index (CI), serves as the numerical signal of the DNB method:


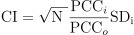


*N* represents the number of molecules in the dominant group or DNB, SD*_i_* denotes the average standard deviation of all molecules in the dominant group, PCC*_i_* signifies the average PCC of all molecule pairs in the dominant group (absolute value), and PCC*_o_* indicates the average PCC of molecule pairs between the dominant group and others. A significant increase in the CI during the measured periods is interpreted as the biological system reaching a critical period or tipping point.

The prioritization of DNB genes was executed based on a structured set of criteria, which are delineated into four priorities: (1) The DNB genes were assessed for their importance at the network level, represented by a percentage or ratio of differentially expressed genes (DEGs) to its adjacent genes; (2) DNB genes were ranked according to their functional roles in the biological pathway; (3) Determining whether a DNB gene is part of the DEGs was based on assigning a value of 1 (indicating “yes”) or 0 (indicating “no”); (4) Determining whether a DNB gene belongs to one of the nine clusters (with a value of 1 for “yes” and 0 for “no”). Additionally, it provided information about the specific cluster to which this DNB gene belongs [13].

### Quantitative real-time polymerase chain reaction (q-PCR)

The extraction of total RNA was performed following the instructions provided with the TRIzol reagent, and the RNA was then reverse transcribed using transcriptase from Invitrogen, USA. The q-PCR was conducted using an SYBR green-based assay (Promega, USA) on a LightCycler^®^ 480 II system (Roche, Germany). β-actin was used as an internal control. The cycling conditions included an initial denaturation at 95 ^∘^C for 5 min, followed by 45 cycles of denaturation at 95 ^∘^C for 10 s, annealing at 57 ^∘^C for 10 s, and extension at 72 ^∘^C for 10 s. This was concluded with a final extension phase at 72 ^∘^C for 10 min. The relative mRNA expression levels were calculated based on the threshold cycle (Ct) values, which were corrected through normalization to β-actin expression, generated using the 2^−ΔΔCt^ equation. The primers used in the assays were as follows: for C1QTNF1, the forward primer was: 5′-GCCTCTACTTCTTCAGCCTCAACG-3′, and the reverse primer was: 5′-GCGAACAAGATCACCACCTCCTC-3′; for β-actin, the forward primer was: 5′-ACATCTGCTGGAAGGTGGAC-3′, and the reverse primer was: 5′-GCACCCAGCACAATGAAGAT-3′.

### Ethical statement

The study received approval from the Ethics Committee of the Third Affiliated Hospital of Sun Yat-sen University (ethics number: 02-057-01). All participating patients provided their consent by signing an informed consent form. The animal use protocols were reviewed and approved by the Laboratory Animal Center of South China Agricultural University (ethics number: 2022DI53).

### Statistical analysis

The distribution of C1QTNF1 gene expression in tumor and normal tissues was analyzed using the Wilcoxon test. To generate Kaplan–Meier curves, along with *P* values and hazard ratios (HRs) with 95% confidence intervals, the log-rank test and univariate Cox proportional hazards regression were employed. The analytical methods and the use of R packages were carried out using R (Foundation for Statistical Computing, 2020) version 4.0.3. A *P* value < 0.05 was deemed to indicate statistical significance.

## Results

### Dynamic gene expression changes in the primary tumor during progression in spontaneous HCC models

**Figure 1.**
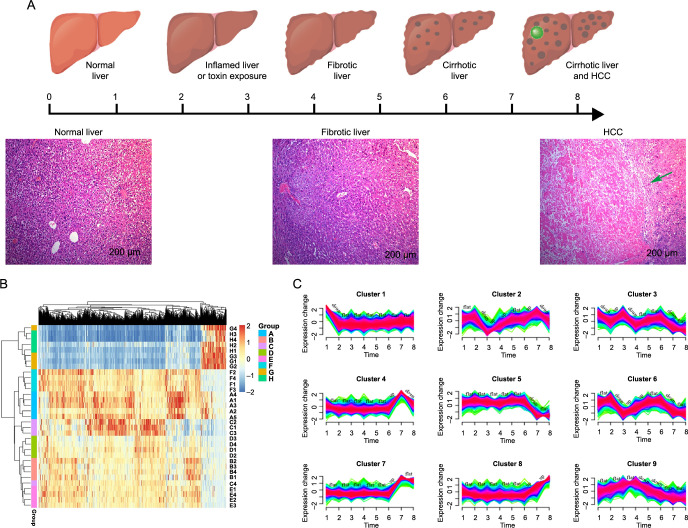
**Dynamic gene expression changes in primary tumors during the progression in spontaneous HCC models.** (A) Depicting the process of establishing the spontaneous HCC mouse model. Mice received intraperitoneal injections of DEN (30 mg/kg) to induce the subcutaneous xenograft model. Liver tissues were collected monthly, and sections were stained with H&E. The spontaneous tumor (indicated by a green arrow) was observed in the 8th month; magnification ×200. (B) Heatmap illustrating unsupervised hierarchical clustering analysis based on DEGs. Gene expression patterns from liver tissues in the 6th month post-DEN injection did not cluster with other time points, suggesting a distinct profile at this stage. (C) Diagram series showcasing the dynamic patterns of DEG changes throughout the development of the spontaneous tumor. HCC: Hepatocellular carcinoma; DEN: N-nitrosodiethylamine; H&E: Hematoxylin and eosin; DEGs: Differentially expressed genes.

Mice were injected with DEN to simulate the development and formation of HCC. Mouse livers were collected monthly, with spontaneous tumors observed in the liver eight months after the DEN injection. The progression from a normal liver to HCC involved several stages, as demonstrated by hematoxylin and eosin (H&E) staining of liver sections: inflammation, fibrosis, and cirrhosis (Figure 1A). The RNA sequencing (RNA-seq) profile was expected to reflect the current state of the tumor. We identified 19,113 DEGs through multiple comparisons with the false discovery rate (FDR) adjustment (*P* < 0.05). Subsequent unsupervised hierarchical clustering of the DEGs allowed the categorization of all liver samples from the eight time points (groups A–H: months 1–8) into two distinct groups (Figure 1B). To further examine the dynamic changes in gene expression during the HCC formation process, we classified all DEGs into nine patterns (cluster 1 to cluster 9) using Mfuzz [23, 24] (Figure 1C). Notably, the majority of gene clusters showed significant downregulation or upregulation primarily in the second and sixth months, respectively.

### C1QTNF1 ranked as one of the core DNBs in HCC development

**Figure 2.**
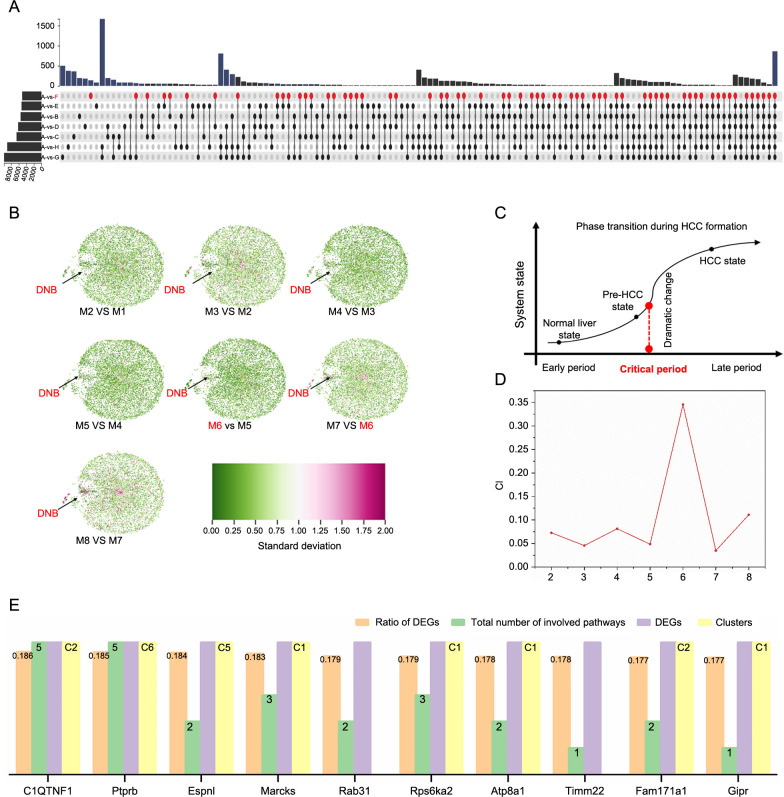
**Prioritizing of C1QTNF1 as one of the core DNBs in HCC development.** (A) An UpSet plot depicting the overlap of gene expression patterns each month with that of the first month, highlighting the sixth month as the point of most significant differential expression; (B) Network diagrams illustrating the dynamic changes in gene expression and network structure characteristic of HCC development beginning in the sixth month; (C) A schematic representation of the phase transition during HCC development, segmented into three stages: normal liver, pre-HCC stage, and HCC stage; (D) Graph indicating that the critical transition occurs at six months post-DEN injection, as inferred from the CI calculated from gene expression data across all time points; (E) A bar graph presenting the ranking of DNB genes based on four prioritized criteria during HCC progression, with C1QTNF1 emerging as the leading candidate for subsequent functional analyses. C1QTNF1: C1q and tumor necrosis factor (TNF)-related 1; DNBs: Dynamic network biomarkers; HCC: Hepatocellular carcinoma; DEN: N-nitrosodiethylamine; CI: Criticality index; M: Month; DEGs: Differentially expressed genes.

We concluded that disease progression did not follow a gradual and monotonic trajectory but rather underwent nonlinear and drastic transitions at certain times. Firstly, we compared the gene expression pattern of each month against that of the first month and employed an UpSet plot to illustrate the intersecting sets. The A vs F comparison showed the smallest overlap, indicating the most significant divergence in gene expression patterns occurred in the 6th month (Figure 2A). Unlike traditional static biomarkers used for disease diagnosis, the DNB serves as a dynamic biomarker capable of predicting diseases. We compared pairwise genes at two consecutive time points and found that this group of DNB genes could significantly signal the tipping point at the sixth month. A network showed the dynamics of the DNB during the HCC development process in spontaneous tumors, based on the SDs of gene expression and the PCCs (Figure 2B). These observations suggest that the tipping point for the development of spontaneous tumors in mice likely occurs at the sixth month. According to the nonlinear dynamic theory, the DNB implied the tipping point as the critical transition before HCC formation (Figure 2C). Upon analysis of RNA-seq expression profiles from tumor samples obtained from orthotopic xenograft mice at eight sequential time points, a pronounced signal for the critical state before tumor formation was detected by the CI at the sixth month post-DEN injection (Figure 2D).

To further understand the underlying roles of DNB genes in the initiation of metastasis in primary tumor cells, we prioritized DNB genes based on four criteria, including the ratio of DEGs, the total number of pathways involved, DEGs, and clusters. C1QTNF1 emerged as the top candidate for further research (Figure 2E).

### CIQTNF1 is an HCC invasion suppressor in vitro and in vivo

**Figure 3.**
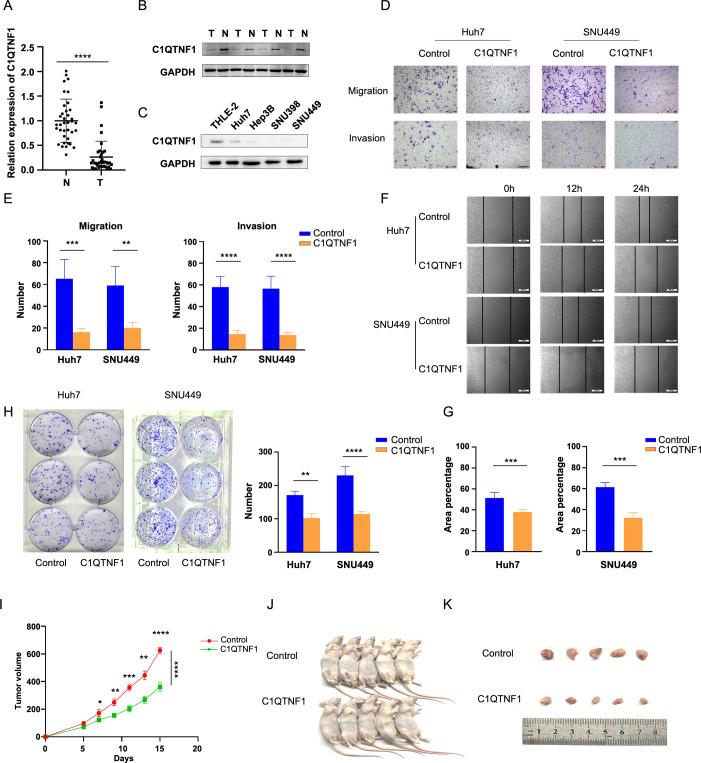
**CIQTNF1 is an HCC invasion suppressor in vitro and in vivo.** (A) q-PCR detecting the C1QTNF1 mRNA expression in tumor and adjacent non-tumor tissues from 37 HCC patients; (B) Western blot analysis depicting the C1QTNF1 protein expression levels in HCC tumor and non-tumor tissue samples; (C) Western blot analysis depicting the C1QTNF1 protein expression levels in a normal liver cell line (THLE-2) and hepatoma cell lines (Huh7, Hep3B, SNU398, SNU449); (D and E) Transwell migration and invasion assays demonstrating the effects of C1QTNF1 overexpression vs control on the chemotactic behavior of Huh7 and SNU449 hepatoma cell lines under monolayer conditions; magnification ×200; (F and G) The scratch assay evaluating the migratory response into wound areas by Huh7 and SNU449 cells with C1QTNF1 overexpression; (H) The clonogenic assay assessing the proliferation ability of Huh7 and SNU449 hepatoma cells upon C1QTNF1 overexpression; (I) Tumor volume comparison in nude mice injected in the inguinal region with SNU449 cells transfected with either scramble control or C1QTNF1-overexpressing lentivirus; (J and K) Tumors harvested from nude mice injected with SNU449 cells transfected with scramble control or C1QTNF1-overexpressing lentivirus after 16 days. * *P* < 0.05; **P < 0.01; ***P < 0.005; ****P < 0.0001. C1QTNF1: C1q and tumor necrosis factor (TNF)-related 1; HCC: Hepatocellular carcinoma; q-PCR: Quantitative real-time polymerase chain reaction; mRNA: Messenger RNA; T: Tumor; N: Non-tumor tissue; GAPDH: Glyceraldehyde 3-phosphate dehydrogenase.

To delve deeper into the role of CIQTNF1 in HCC, we performed a series of studies to investigate the function of C1QTNF on oncogenic behaviors of hepatoma cells, including their growth, migration, and invasion. Firstly, we assessed the mRNA expression of C1QTNF1 in both tumor and adjacent non-tumor tissue samples from 37 HCC patients. The results revealed that C1QTNF1 mRNA expression levels in tumor tissues were lower compared to those in non-tumor tissues (Figure 3A). The protein expression levels of C1QTNF1 were consistent with the mRNA expression results (Figure 3B).

We then investigated the association between C1QTNF1 expression and its functional roles in HCC cells. We examined the expression profile of C1QTNF1 in the human hepatocyte cell line THLE-2 and four hepatoma cell lines (Huh7, Hep3B, SNU398, and SNU449). Compared with THLE-2 cells, C1QTNF1 was found to be significantly downregulated in all hepatoma cell lines (Figure 3C). To further verify the role of C1QTNF1 as a tumor suppressor, we induced overexpression of C1QTNF1 in Huh7 and SNU449 cells using a lentiviral vector. The results of cell migration and invasion assays, alongside scratch assays, demonstrated that cells overexpressing C1QTNF1 exhibited decreased migration and invasion abilities in comparison to the control group (Figure 3D–3G). Furthermore, clonogenic assays revealed that overexpression of C1QTNF1 significantly inhibited the growth of both Huh7 and SNU449 cell lines (Figure 3H).

To confirm the observations in vivo, a subcutaneous tumor model was established in nude mice using C1QTNF1-overexpressing SNU449 cells (Figure 3I). Small tumors were visible by the 6th day, and from that point, the tumor volume was measured every other day. The group with inducible C1QTNF1 expression developed smaller tumor volumes compared to the control group (Figure 3J and 3K).

### Rewiring of C1QTNF1-subnet before the HCC formation tipping point

**Figure 4.**
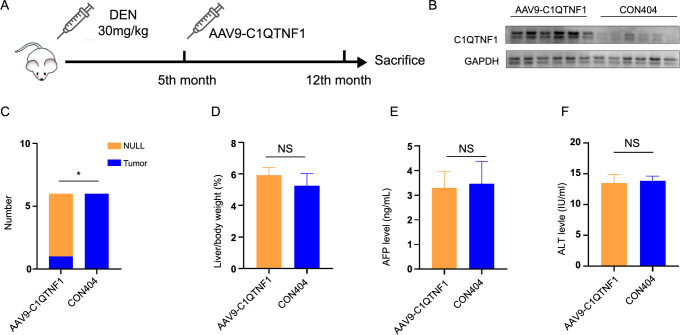
**Rewiring of C1QTNF1-subnet before the HCC formation tipping point.** (A) Depicting a spontaneous HCC mouse model establishment. At the fifth month, AAV9-C1QTNF1 was administered to induce C1QTNF1 overexpression in the liver, with CON404 serving as the control, both delivered through the tail vein. The livers were harvested after 12 months. (B) Western blot analysis assessing C1QTNF1 expression in the livers of mice from both the AAV9-C1QTNF1 and CON404 groups. (C–F) Graphs depicting the assessment of tumor development (C), liver/body weight ratio (D), serum AFP levels (E), and serum ALT levels (F), at 12 months post-DEN injection. *P < 0.05. C1QTNF1: C1q and tumor necrosis factor (TNF)-related 1; HCC: Hepatocellular carcinoma; AAV9: Adeno-associated virus 9; CON: Control; AFP: Alpha-fetoprotein; ALT: Alanine aminotransferase; DEN: N-nitrosodiethylamine; GAPDH: Glyceraldehyde 3-phosphate dehydrogenase; NULL: No tumor was observed; NS: No significance.

Since tumor formation is potentially reversible at the tipping point, we utilized adeno-associated virus 9 to deliver C1QTNF1 (AAV9-C1QTNF1) mRNA sequence and induce overexpression of C1QTNF1. A control virus (CON404) was administered as a comparison. Both were injected via the tail vein following the fifth-month post-DEN injection, and livers were collected in the twelfth month for analysis (Figure 4A). The efficacy of AAV9-mediated transfection is depicted in Figure 4B. As illustrated in Figure 4C, AAV9-C1QTNF1 administration was effective in preventing HCC formation. The liver characteristics of the treated mice closely resembled those of the control mice (Figure 4D–4F).

### The prognostic and diagnostic function of C1QTNF1 in HCC patients

**Figure 5.**
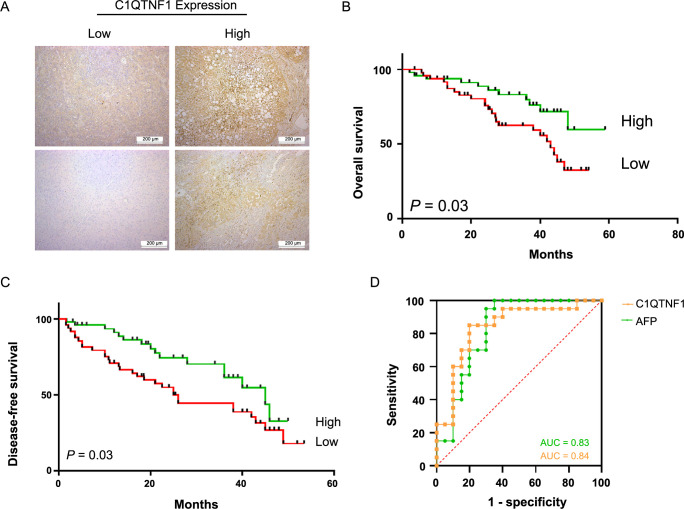
**Assessment of the prognostic and diagnostic value of C1QTNF1 in HCC patients.** (A) Immunohistochemical staining of C1QTNF1 in 96 HCC tissue samples; magnification ×200. (B and C) Kaplan–Meier survival curves displaying the OS (B) and DFS (C) based on C1QTNF1 expression levels measured using the immunohistological staining score. Group differences were evaluated using the log-rank test (*P* ═ 0.03 for both). (D) ROC curve analysis comparing the diagnostic value of C1QTNF1 levels in HCC tissues with serum AFP levels. The AUC for C1QTNF1 and AFP were 0.84 and 0.83, respectively. C1QTNF1: C1q and tumor necrosis factor (TNF)-related 1; HCC: Hepatocellular carcinoma; OS: Overall survival; DFS: Disease-free survival; ROC: Receiver operating characteristic; AFP: Alpha-fetoprotein; AUC: Area under the curve.

To explore the prognostic value of C1QTNF1, we initially evaluated its expression in tumor tissues from 96 HCC patients using immunohistochemical staining (Figure 5A), with patient characteristics detailed in the supplementary data. Patients who exhibited high C1QTNF1 expression had better overall survival (OS) and disease-free survival (DFS) (Figure 5B and 5C). Additionally, lower C1QTNF1 expression was observed in more advanced tumor stages, as outlined in the supplementary data. To further determine C1QTNF1’s potential as a differential diagnostic biomarker, we measured serum concentrations of C1QTNF1 in both HCC and CHB patients through ELISA. The analysis showed that the area under the curve (AUC) for C1QTNF1 (0.84) was comparable to that of AFP (0.83) (Figure 5D).

### Dynamic gene changes associated with C1QTNF1 during HCC progression

**Figure 6.**
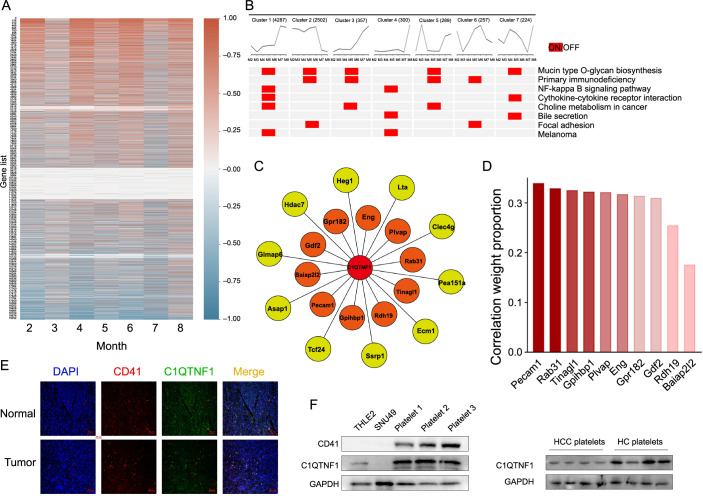
**Dynamic gene changes associated with C1QTNF1 during HCC progression.** (A) Heatmap displaying gene dynamics associated with C1QTNF1 during HCC progression; (B) Heatmap illustrating the dynamic activity of related KEGG pathways involving C1QTNF1 and its correlated DEGs; (C) Diagram depicting the top 20 most relevant genes identified each month, based on an overall final ranking across the timeline from the first to the eighth month; (D) Graph illustrating the top ten genes and their proportion in all correlation weights around the tipping point; (E) Immunofluorescent staining displaying the correlation of C1QTNF1 (green fluorescence) and CD41 (red fluorescence), a platelet marker, in both normal and tumor tissues; magnification ×200; (F) Western blot analysis comparing C1QTNF1 expression in THLE-2 and SNU449 cell lines and platelets isolated from healthy donors. Furthermore, C1QTNF1 expression was compared between platelets from HCC patients and healthy controls. C1QTNF1: C1q and tumor necrosis factor (TNF)-related 1; HCC: Hepatocellular carcinoma; KEGG: Kyoto Encyclopedia of Genes and Genomes; DEGs: Differentially expressed genes; CD: Cluster of differentiation; NF: Nuclear factor; Pecam1: Platelet and endothelial cell adhesion molecule 1; DAPI: 4′, 6-diamidino-2-phenylindole; GAPDH: Glyceraldehyde 3-phosphate dehydrogenase; HC: Healthy controls.

Next, in examining the relationship between the initiation of biological functions enriched by C1QTNF1 and its inverse DEGs before and after the critical period of HCC formation initiation, we observed enrichment in most typical cancer-related pathways. Firstly, we calculated the most relevant gene for each month to generate an overall final ranking (Figure 6A). Then, we examined the relationship between the pathways and biological functions enriched by C1QTNF1 (Figure 6B), analyzing the top ten genes and their proportions in all correlation weights around the tipping point (5th and 6th months). Pearson correlation analysis was used to determine the top 20 genes (Figure 6C). By focusing on the correlation weight proportion during the tipping time, we identified platelet and endothelial cell adhesion molecule 1 (*Pecam1*) as the most related gene (Figure 6D). The protein encoded by *Pecam1* is found on the surface of platelets, monocytes, neutrophils, and some types of T cells and is involved in endothelial cell intercellular junctions [25]. Integrating the gene correlation and pathway enrichment findings led us to postulate that C1QTNF1 is predominantly expressed in platelets. The confocal microscopy results showed that C1QTNF1^+^ cells and CD41^+^ cells were located in the same region (Figure 6E). We then assessed C1QTNF1 expression in the THLE-2 cell line, SNU449 cells, and platelets isolated from healthy donors to verify our hypothesis. The findings revealed that C1QTNF1 expression is higher in platelets from HCC patients compared to those from healthy controls (Figure 6F), suggesting a role for C1QTNF1 in platelet-related cancer signaling pathways.

## Discussion

HCC is a serious global health problem, exerting a substantial burden on the global medical system. Projections estimate that over one million individuals will be affected by HCC annually by the year 2025 [1, 26, 27]. Despite recent improvements in diagnostic and treatment approaches, the prognosis of patients with advanced HCC remains poor. Therefore, it is of paramount importance to search for more reliable preventive strategies for HCC [28].

The DNB method has been previously used to identify biomarkers of pre-exhausted CD8+ T cells in colorectal cancer and for the tipping point in HCC pulmonary metastasis [29]. Continuous progress has been witnessed in the DNB method due to the recent researchers’ efforts. The single-sample landscape entropy (SLE) method was developed to identify the tipping point during disease progression using data from just a single sample [12]. Additionally, a model-free approach known as the directed-network rank score (DNRS) has been introduced to detect early-warning signals of critical transitions in complex biological systems [17]. It is really exhilarating that the tipping point detector (TPD), a web tool for the detection of the tipping point during the dynamic process of biological systems, is now online [30]. The development of HCC is a progressive and complex process involving inflammation, fibrosis, and cirrhosis. The existing static biomarker for HCC, such as AFP, heat shock protein 70 (HSP70), and glypican-3 (GPC3), may not always accurately reflect the critical transitions. By establishing a DNB model in our study, we have identified that C1QTNF1 played a crucial role at the tipping point of HCC formation.

C1QTNF1 mRNA is mainly expressed in adipose tissue and is also found in the heart, placenta, liver, muscles, and kidneys. It has been shown to inhibit cardiac hypertrophy and fibrosis by activating the AMP-activated protein kinase alpha (AMPKα) [31]. The circulating level of C1QTNF1 was found to be increased in nonalcoholic fatty liver disease (NAFLD) and associated with various metabolic parameters [32, 33]. In this study, we validated the suppressive effect of C1QTNF1 on HCC. In both cell and mouse models, overexpression of C1QTNF1 can significantly suppress tumor growth and reduce the invasive and migratory capabilities of the tumor. More importantly, overexpression of C1QTNF1 at the tipping time point can effectively inhibit spontaneous HCC formation in mice. We evaluated the gene encoding C1QTNF1 and identified its expression in platelets. A study showed that C1QTNF1 can block platelet activation and aggregation by binding to fibrillar collagen type I [34].

Recent studies have revealed the significant role of platelets in the pathogenesis of a variety of vascular inflammatory processes, including autoimmune diseases, viral infections, sepsis, and the growth and metastasis of many types of tumors [35, 36]. Consequently, platelets are increasingly recognized as immune cells that contribute to all stages of the immune response [37]. The interaction between platelets and immune cells is mediated through the platelet P-selectin (CD62P) and the neutrophil P-selectin glycoprotein ligand-1 (PSGL-1) [38]. Circulating neutrophils identify and engage with activated platelets at sites of endothelial damage, which is essential for the migration of neutrophils and the execution of their immune effector functions at sites of tissue injury [39]. Additionally, platelet-derived serotonin has been shown to promote liver regeneration in mice following partial liver resection [40].

Our study also has certain limitations. The mouse model is a widely used and effective tool for studying spontaneous HCC, allowing for dynamic tracking by researchers. However, it is difficult to completely eliminate the species differences between mice and humans. In addition, the human HCC exhibits significant heterogeneity. Consequently, findings obtained from mouse models must be validated with human samples, a process many researchers are currently pursuing. Additionally, the DNB method emphasizes dynamic changes, underscoring the importance of temporal data. Future research, within ethical limits, might analyze larger human samples over extended periods. For instance, conducting long-term dynamic analyses of hepatitis B patients could more accurately reflect the transition from hepatitis to liver cancer. Such longitudinal studies could vastly improve our ability to identify individuals at high risk of developing HCC.

Predicting tumor onset is critical for the early diagnosis of HCC, as the prognosis for patients with advanced stages of the disease remains unfavorable. In the initial stages of HCC, surgical resection is the primary treatment option. However, for advanced or unrespectable tumors, more comprehensive approaches including surgery, chemotherapy, and immunotherapy are utilized [3, 41, 42]. The use of conventional HCC therapies is currently being challenged by the advent of new systemic therapies, including immune checkpoint inhibitors (ICIs), tyrosine kinase inhibitors (TKIs), and monoclonal antibodies [43]. While numerous therapeutic strategies have shown promising efficacy in animal models, their clinical application faces multiple challenges. Furthermore, there is a continuous need for the identification of new targets and the enhancement of clinical treatment outcomes. The DNB method holds the potential for uncovering new diagnostic biomarkers that could signal the point before the onset of tumor development or metastasis. We believe that advancements in technology and models are expected to shed light on the underlying mechanisms of these processes. In conclusion, our study contributes new perspectives on the research of HCC development and lays the groundwork for novel treatments that target the pivotal tipping points preceding tumor formation.

## Conclusion

C1QTNF1 has been identified as the predominant DNB gene at the critical tipping point in the development of HCC. It acts as a core suppressor of HCC invasion and metastasis. Furthermore, C1QTNF1 presents a novel therapeutic target in HCC treatment, operating through the platelet-related cancer signaling pathway.

## Supplemental data

**Table S1:** Patient characteristics

**Variable**	**C1QTNF1 expression**		**Total** **(*n* ═ 96)**	***P* value**
	**High**	**Low**		
Age (years)	51.0 ± 11.1	50.0 ± 10.3	50.3 ± 10.8	0.6136
Gender				> 0.9
Male	43	44	87	
Female	5	4	9	
Hepatitis virus				0.700
HBV (+)	45	47	92	
HCV (+)	3	0	3	
HBV (+) HCV (+)	0	1	1	
HBV-DNA				0.678
< 1 × e^2^	4	2	6	
> 1 × e^2^	44	43	87	
TNM Stage				0.016
I	42	11	53	
II	6	4	10	
III	0	32	32	
IV	1	0	1	
Multiplicity				> 0.9
Solitary	46	47	93	
Multiple	2	1	3	
Differentiation				0.0568
I	6	2	8	
II	33	43	76	
III	10	0	10	
IV	2	0	2	
Tumor Size (cm)	3.6 ± 1.5	5.5 ± 2.9	4.6 ± 2.48	< 0.001
MVI				0.7589
No	43	41	84	
Yes	5	7	12	
AFP (ng/mL)				0.0376
<400	40	30	70	
>400	8	18	26	

Data are presented as mean ± standard deviation or counts. C1QTNF1: C1q and tumor necrosis factor (TNF)-related 1; HBV: Hepatitis B virus; HCV: Hepatitis C viruls; TNM: Tumor, node, metastases; MVI: Microvascular invasion; AFP: Alpha-fetoprotein.

## Data Availability

The data supporting the findings of this study are available from the corresponding authors upon reasonable request.
